# *Clostridium massiliamazoniense* sp. nov., New Bacterial Species Isolated from Stool Sample of a Volunteer Brazilian

**DOI:** 10.1007/s00284-020-02099-9

**Published:** 2020-07-01

**Authors:** Niokhor Dione, Cheikh Ibrahima Lo, Didier Raoult, Florence Fenollar, Pierre-Edouard Fournier

**Affiliations:** 1 Aix Marseille Univ, IRD, AP-HM, MEФI, Marseille, France; 2 IHU-Méditerranée Infection, 19-21 Boulevard Jean Moulin, 13385 Marseille Cedex 05, France; 3 Aix Marseille Univ, IRD, AP-HM, SSA, VITROME, Marseille, France

## Abstract

The study of the gut microbiota by the “culturomics concept” permitted us to isolate, from human stool sample, an unknown anaerobic bacterium within the genus *Clostridium* for which we propose the name *Clostridium massiliamazoniense* sp. nov. It was isolated from the fecal flora of a healthy 49-year-old Brazilian male. Here, we describe the characteristics of this organism and its complete genome sequencing and annotation*. Clostridium massiliamazoniense* sp. nov., ND2^T^ (= CSURP1360 = DSMZ 27309) is a Gram-positive, obligate anaerobic member of *Firmicutes* with a 3,732,600 bp-long genome and a G+C content of 27.6%.

## Introduction

Characterization of bacteria by mass spectrometry and high-throughput genome sequencing has allowed improving their proteomic and genetic characterization [1]. Therefore, it was proposed to include these data into the taxono-genomic description of new bacterial taxa [2, 3]. This strategy combines the analysis of the complete genome sequence, MALDI-TOF spectrum, and main phenotypic features of any bacterium of interest [4].

The genus *Clostridium* (Prazmowski, 1880) was created in 1880. It is classified within the phylum *Firmicutes*. It contains anaerobic rod-shaped bacilli that are able to produce endospores. Members of the *Clostridium* genus are essentially environmental bacteria or belong to the gut flora of mammals. However, several species are major human pathogens, including *C. botulinum*, *C. difficile*, *C. tetani*, and *C. perfringens* [5, 6].In addition, some species, such as *C. butyricum* and *C. pasteurianum*, are able to fix nitrogen and have agricultural and industrial applications [7, 8]. Herein, we describe a new bacterial species, *Clostridium massiliamazoniense* sp. nov., ND2^T^ using the taxono-genomics approach.

## Material and Methods

### Organism Informations and Collection

A stool sample was collected from a 49-year-old volunteer and healthy male living in Brazil. The patient gave an informed and signed consent, and the agreement of the ethics committee of the Institut Fédératif de Recherche 48 (Aix-Marseille University, Marseille, France) was obtained under number 09-022. At the time of sampling, the patient was not under antibiotics. The stool sample was stored at − 80 °C until further investigation.

### Strain Isolation and Identification by MALDI-TOF MS and 16S rRNA Sequencing

Strain ND2 was isolated from stool in November 2013 by culture on 5% sheep blood-enriched Columbia agar (bioMérieux, Marcy l'Etoile, France) at 37 °C in anaerobic atmosphere after an initial thermal shock (15 min at 65 °C for in a dry bath) followed by 3 days of stool sample pre-incubation in an anaerobic blood culture bottle enriched with 5% sheep blood (Thermo Fisher Scientific, Waltham, USA) and 5% bovine rumen fluid (obtained from a local abattoir) filtered on a 0.2 µm filter.

Strain ND2 was screened by MALDI-TOF MS using a Microflex spectrometer (Bruker Daltonics, Leipzig, Germany), as previously described [9]. Eighteen distinct deposits were made on MTP 384 MALDI-TOF target plate for strain ND2, from eighteen isolated colonies. Spectra were recorded in the positive linear mode for the mass range of 2000 to 20,000 Da (parameter settings: ion source 1 (IS1), 20 kV; IS2, 18.5 kV; lens, 7 kV). A spectrum was obtained after 675 shots at a variable laser power. The eighteen spectra were imported into the MALDI BioTyper software (version 3.0, Bruker Daltonics) and analyzed by standard pattern matching (with default parameter settings) against the main spectra of 5625 bacteria including 216 spectra from *Clostridiu*m species with validly published names that are part of the reference data contained in the BioTyper database. An isolate is considered correctly identified by MALDI-TOF MS at the species level if it has a score of > 1.9 or at the genus level if it only has a score > 1.7. The spectra obtained for strain ND2 were added to our database.

The spectrum of strain ND2 did not match those of any species in the Bruker database. Therefore, we sequenced its 16S rRNA gene as previously described [10].

### Phenotypic Characteristics

#### Culture Conditions

Different growth temperatures (25, 30, 37, 45 °C) were tested. The growth of strain ND2 was also tested on 5% sheep blood-enriched Columbia agar (bioMerieux) in aerobic atmosphere, with or without 5% CO_2_, and under anaerobic and microaerophilic conditions using GENbag anaer and GENbag microaer systems (bioMérieux), respectively.

#### Biochemical, Sporulation, and Motility Assays

The biochemical characteristics of strain ND2 were studied using the API Rapid 32A, API ZYM and API 50 CH strips (bioMérieux).The ability to form spores was tested following a thermal shock and the motility assay was performed by direct examination of a fresh colony using a DM 1000 optical microscope (Leica, Nanterre, France) at a × 400 magnification.

#### Antibiotic Susceptibility

The susceptibility of strain ND2 to antimicrobial agents was tested using the disk diffusion method [11]. We tested the following antibiotics: amoxicillin, amoxicillin/clavulanic acid, imipenem clindamycin, doxycycline, rifampicin, tetronidazole, trimethoprim/sulfamethoxazole, and vancomycin.

#### Transmission Electron Microscopy

A 3.5 µL drop of bacterial suspension was applied for 30 s to the top of a formvar carbon 400 mesh nickel grid (FCF400-Ni, EMS) that was previously glow discharged. After drying using filter paper, bacteria were immediately stained with 1% ammonium molybdate for 1 sec. Electron micrographs were acquired on a Tecnai G20 transmission electron microscope (FEI Company, Limeil-Brevannes, France) operated at 200 keV.

#### FAME Analysis by GC/MS

Three samples were prepared with approximately 50 mg of bacterial biomass harvested from several culture plates. Cellular fatty acid methyl esters were prepared as described by Sasser [12]. GC/MS analyses were carried out as described elsewhere [13].

### Genomic DNA Preparation

Strain ND2 was cultivated on 5% sheep blood-enriched Columbia agar (bioMérieux, Marcy l’Etoile, France) at 37 °C in anaerobic condition. After 24 h of incubation, colonies grown on three culture plates were collected and suspended in 4 × 100 µL of TE buffer. For cell lysis, 200 µL of this suspension and 2.5 µg/µL lysozyme were added in 1 mL TE buffer and incubated during 30 min at 37 °C. Then, 20 µg/µL proteinase K was added for an overnight incubation at 37 °C. Extracted genomic DNA (gDNA) was then purified as previously described by Lo et al. [17].

### Genome Sequencing and Assembly

A MiSeq sequencer (Illumina, San Diego, CA, USA) was used for sequencing the genomic DNA (gDNA) of strain ND2. The gDNA was barcoded and pooled with 11 other projects with the Nextera Mate-Pair sample prep kit (Illumina). The Mate-Pair library was prepared with 1 µg of gDNA using the Nextera Mate-Pair Illumina guide, and the gDNA sample was simultaneously fragmented and tagged with a Mate-Pair junction adapter. The fragmentation pattern of gDNA was validated on an Agilent 2100 BioAnalyzer (Agilent Technologies, Santa Clara, CA, USA) with a DNA 7500 labchip. The sequencing run was performed as reported in previous studies [14–18]. Then, the obtained reads were trimmed and assembled using the CLC genomics Workbench (CLCbio, Seoul, Korea).

### Genome Annotation and Analysis

The online website Prodigal was used to detect Open Reading Frames (ORFs) (https://prodigal.ornl.gov/). The predicted bacterial protein sequences were identified by comparison with the GenBank [19] and Clusters of Orthologous Groups (COG) databases using BLASTP. The RNAs, signal peptides and transmembrane helices were detected using tRNAScan-SE [20], RNAmmer [21], SignalP [22] and TMHMM [23], respectively. The presence of mobile genetic structures was revealed using PHAST [24] and RAST [25]. In order to visualize and manage genomic data, Artemis [26] and DNA Plotter [27] were used successively. The Mauve alignment tool (version 2.3.1) [28] enabled performing multiple genomic sequence alignment between strain ND2 and closely related species. Finally, the average nucleotide identity between genomes was evaluated using the GGDC software [29].

## Results

### Phylogenic Analysis

**Fig. 1:**
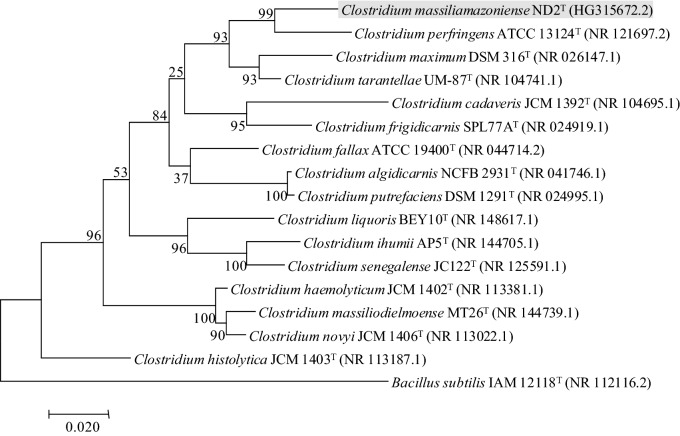
Phylogenetic tree obtained from the comparison of 16S rRNA sequences highlighting the position of *C. massiliamazoniense* ND2T relative to other type strains within the genus *Clostridium*. GenBank accession numbers are indicated in parentheses. Sequences were aligned using CLUSTALW, and phylogenetic inferences were obtained using the maximum-likelihood method within the MEGA software. Numbers at the nodes are percentages of bootstrap values greater than 70% obtained by repeating 1000 times the analysis to generate a majority consensus tree. *Bacillus subtilis* was used as outgroup. The scale bar represents a 2% nucleotide sequence divergence

The MALDI-TOF MS score obtained from strain ND2^T^ colonies were < 1.7 and did not enable any identification at the species level. This strain exhibited a 95.4% nucleotide sequence similarity with *C. perfringens* ATCC 13124^ T^, the phylogenetically closest bacterial species with a validly published name (Fig. 1). This similarity value is lower than the 98.7% threshold used to delineate a new species without carrying out DNA-DNA hybridization [30, 31].

### Phenotypic Description

Among the incubation temperatures tested (25, 30, 37, 45 °C) growth was observed at 37 and 45 °C, with an optimal growth at 37 °C, 24 h after inoculation. At 45 °C, growth was observed but was slower. Growth was only observed in anaerobic atmosphere on 5% sheep blood-enriched Columbia agar (bioMérieux, Marcy l’Etoile, France). Colonies were dark gray with irregular edges and had a diameter of 1.0 mm.

Strain ND2^T^ possessed acid phosphatase, naphthol-AS-BI-phosphohydrolase, and galactosidase. It was able to ferment D-glucose and D-maltose. The phenotypic and biochemical characteristics of strain ND2^T^ were compared with those of other related species (Table 1). It was susceptible to amoxicillin, imipenem, doxycycline, rifampicin, metronidazole, and vancomycin. The major fatty acid was octadecanoic acid (14.4% relative abundance). The other abundant fatty acids were mainly saturated species while minor ones were unsaturated (Supplement Table S2).

**Table 1:** Differential characteristics of *C. massiliamazoniense* ND2^T^ (**1**), *C. dakarense* strain FF1 (**2**), *C. perfringens* DSM 599^T^ (**3**), *C. liquoris* DSM 100320^T^ (**4**)*,* and *C. frigidicarnis* DSM 12271^T^

Properties	1	2	3	4
Cell diameter (µm)	0.9	1.3	0.5–1.0	1.3–1.6
Oxygen requirement	−	−	−	−
Gram stain	+	+	+	+
Motility	−	−	−	+
Endospore formation	+	+	+	+
Indole	+	Na	−	-
Production of				
Acid phosphatase	+	+	Na	Na
Catalase	−	−	−	−
Oxidase	−	Na	Na	−
Nitrate reductase	−	+	Na	−
Urease	−	Na	Na	−
β-galactosidase	+	+	Na	Na
* N*-acetyl-glucosamine	−	Na	Na	−
Acid from				
l-Arabinose	−	−	Na	−
d-Ribose	−	+	Na	Na
d-Mannose	−	+	w	+
d-Mannitol	−	−	+	−
Sucrose	−	+	Na	Na
d-glucose	+	+	+	+
d-fructose	−	+	Na	+
d-maltose	+	+	+	+
d-lactose	−	+	+	−
G+C content (mol%)	27.6	27	34.4	27.3
Habitat	Human gut	Colonic flora	Fermentation pit	Bovine feces

*Na* data not available, *w* weak

### Genome Properties

**Fig. 2:**
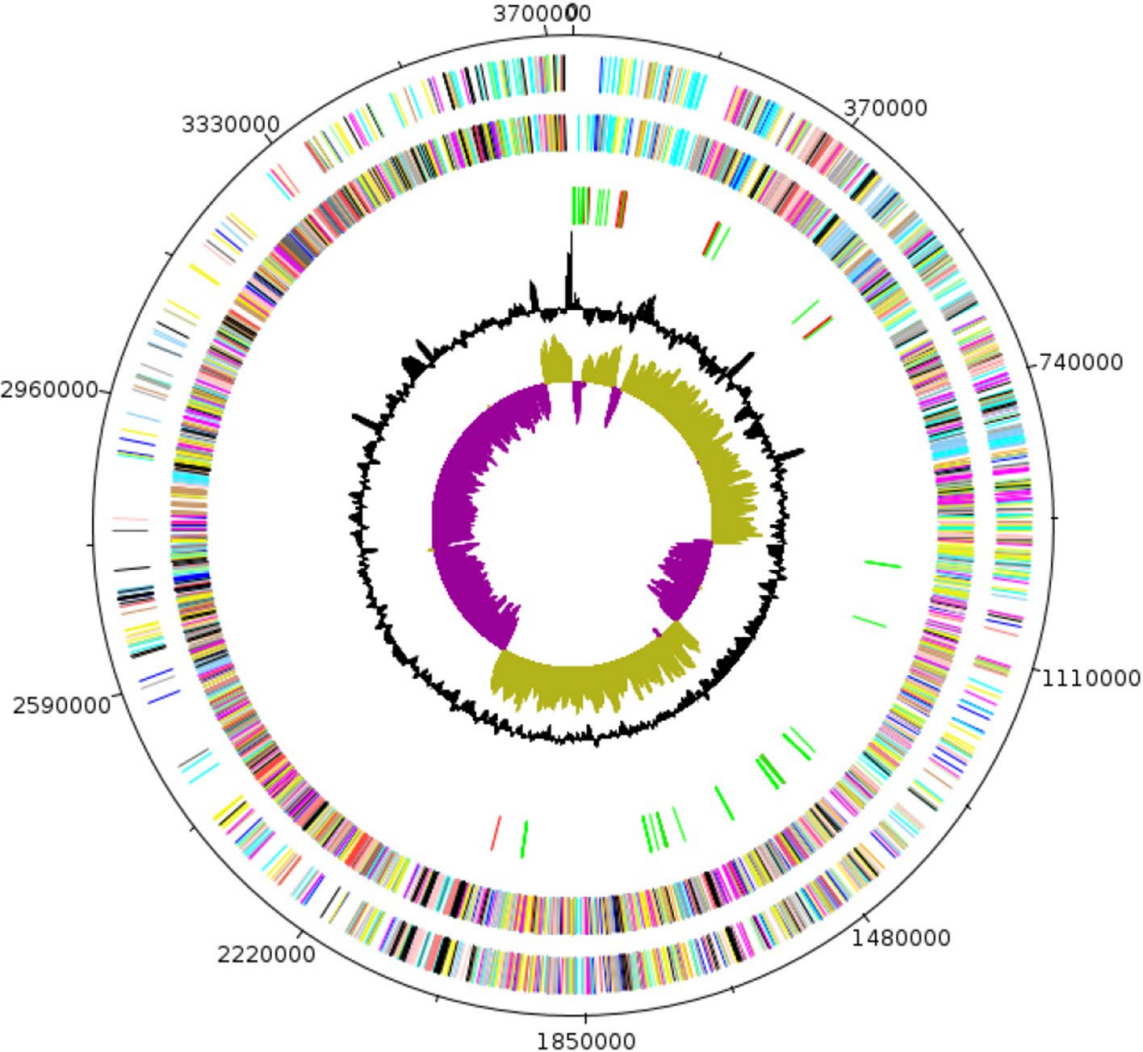
Graphical circular map of *C. massiliamazoniense* ND2^T^chromosome. From outside to the center: genes on the forward strand colored by COG categories (only genes assigned to COG), genes on the reverse strand colored by COG categories (only gene assigned to COG), RNA genes (tRNAs green, rRNAs red), GC content, and GC skew (Color figure online)

The genome was 3,732,600-bp long with a G+C content of 27.6% (Fig. 2). It was composed of 12 contigs. Among the 3518 predicted genes, 3403 were protein-coding genes, and 115 were RNAs (10 genes are 5S rRNA, 11 genes are 16S rRNA, 7 genes are 23S rRNA, 83 genes are tRNA genes and 4 genes are ncRNAs). A total of 2305 genes (65.53%) were assigned a putative function (by COGs or by BLAST against NR), and 397 (11.2%) genes were identified as ORFans. The remaining genes were annotated as hypothetical proteins. The statistics of the genome are detailed in Table 2 while the distribution of genes into COG functional categories is presented in Table 3.

**Table 2:** Nucleotide content and gene count of the genome

Attribute	Genome (total)	
	Value	% of total^a^
Size (bp)	3,732,600	100
G+C content (%)	1,030,197	27.6
Coding region (bp)	3,039,312	81.42
Total genes	3518	100
RNA genes	115	3.26
Protein-coding genes	3403	96.75
Genes with function prediction	2305	65.53
Genes assigned to COGs	2075	58.99
Genes with peptide signals	148	4.20
CRISPR repeats	01	0.02
ORFans genes	397	11.2
Genes with transmembrane helices	546	15.52

^a^The total is based on either the size of the genome in base pairs or the total number of protein-coding genes in the annotated genome

**Table 3:** Number of genes associated with the 25 general COG functional categories

Code	Value	% of total^a^	Description
J	156	4.58	Translation
A	0	0.0	RNA processing and modification
K	223	6.55	Transcription
L	158	4.64	Replication, recombination and repair
B	2	0.06	Chromatin structure and dynamics
D	30	0.88	Cell cycle control, mitosis and meiosis
Y	0	0.0	Nuclear structure
V	66	1.94	Defense mechanisms
T	125	3.67	Signal transduction mechanisms
M	148	4.35	Cell wall/membrane biogenesis
N	55	1.62	Cell motility
Z	0	0.0	Cytoskeleton
W	0	0.0	Extracellular structures
U	41	1.20	Intracellular trafficking and secretion
O	86	2.53	Post-translational modification, protein turnover, chaperones
C	154	4.53	Energy production and conversion
G	170	5.0	Carbohydrate transport and metabolism
E	229	6.73	Amino acid transport and metabolism
F	68	2.0	Nucleotide transport and metabolism
H	103	3.03	Coenzyme transport and metabolism
I	59	1.73	Lipid transport and metabolism
P	155	4.55	Inorganic ion transport and metabolism
Q	28	0.82	Secondary metabolites biosynthesis, transport and catabolism
R	325	9.55	General function prediction only
S	228	6.70	Function unknown
-	230	6.53	Not in COGs

^a^The total is based on the total number of protein-coding genes in the annotated genome

### Genome Comparison

The genome size of *C. massiliamazoniense* ND2^T^ (3.7 Mb) was larger than those of *Clostridium perfringens* ATCC 13124, *Clostridium liquoris* DSM 100320, *Clostridium fallax* DSM 2631, and *Clostridium cadaveris* LH052 (3.2, 2.8, 2.7, and 3.5 Mb, respectively) but smaller than those of *Clostridium frigidicarnis* DSM 12271 (4.3 Mb).

Strain ND2 had dDDH values of 28.6, 31.8, 28.8, 28.4, 32.7, and 32.5 with *C. cadaveris* LH052, *C. fallax* DSM 2631, *C. frigidicarnis* DSM 12271, *C. liquoris* DSM 100320, *C. perfringens* ATCC 13124, and *C. tarantellae* DSM 3997. These values far below the recommended 70% threshold value suggest that the strain ND2 is new bacterial species (Table 4).

**Table 4:** Pairwise comparison of *C. massiliamazoniense* ND2^T^ with *C. cadaveris* LH052 (**Cca**), *C. fallax* DSM 2631 (**Cfa**), *C. frigidicarnis* DSM 12271 (**Cfr**), *C. liquoris* DSM 100320 (**Cli**), *C. perfringens* ATCC 13124 (**Cpe**), and *C. tarantellae* DSM 3997 (**Cta**) using GGDC, formula 2 (DDH estimates based on identities/HSP length)

	Cca	Cfa	Cfr	Cli	Cma	Cpe	Cta
Cca	100%	29.2	30.8	28.6	28.6	29.3	28.9
Cfa		100%	30.2	29.2	31.8	31.8	32.2
Cfr			100%	28.8	28.8	29.5	29.8
Cli				100%	28.4	28.7	28.9
Cma					100%	32.7	32.5
Cpe						100%	33.8
Cta							100%

**Fig. 3:**
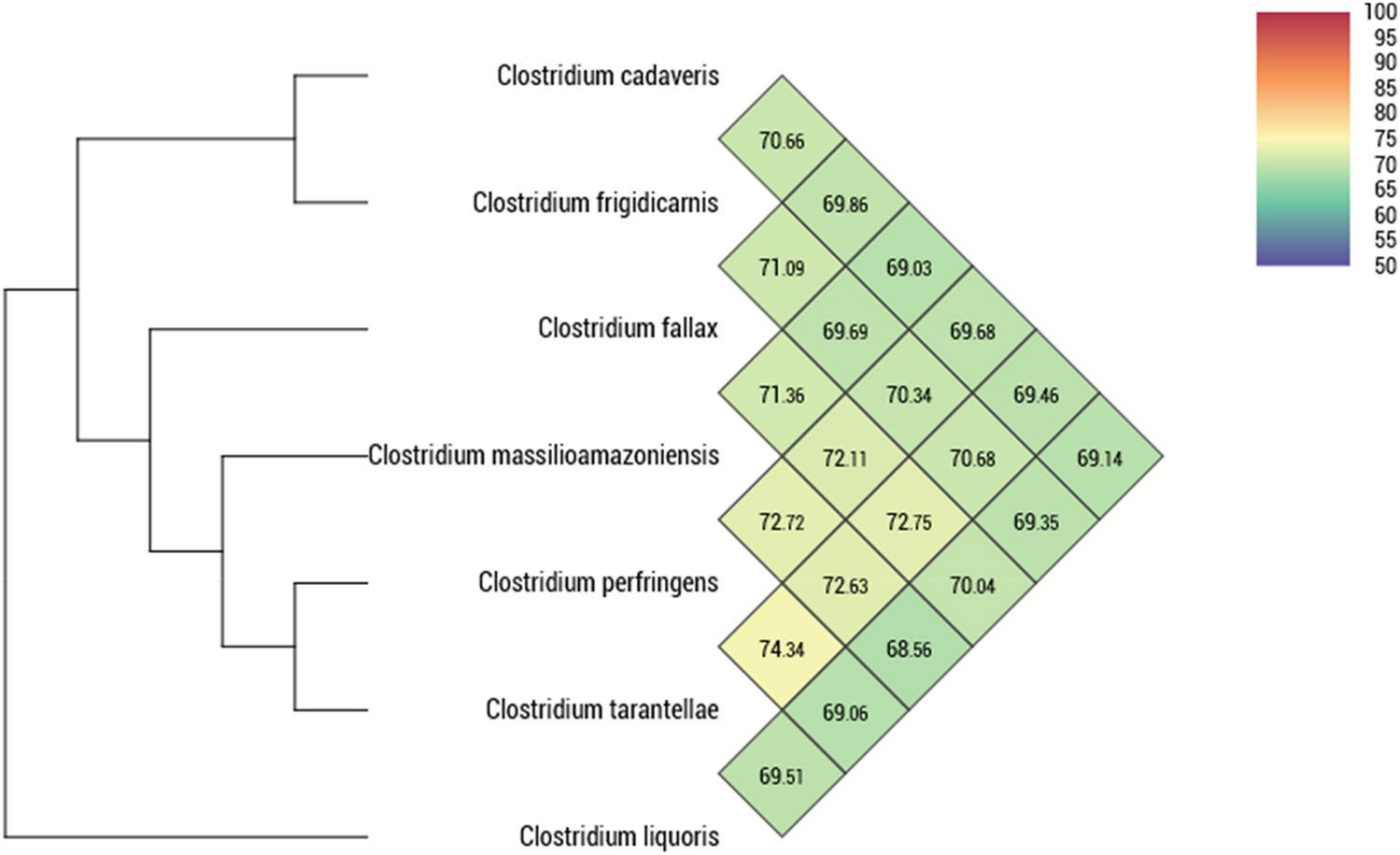
Heatmap generated with OrthoANI values calculated using the OAT software between *Clostridium massilioamazoniensis* sp. nov. and other related *Clostridium* species

The degree of genomic similarity of the strain ND2 compared to its related species was calculated with Orthologous ANI Tool (OAT) software [32]. *C. massiliamazoniense* shared with *C. liquoris* the lowest OrthoANI value (68.56%) and with *C. perfringens* the highest value (72.72%). Analysis of all studied genomes showed that 74.34% was the highest value shared between *C. perfringens* and *C. tarantellae* (Fig. 3).

## Conclusion

We formally propose the creation of *Clostridium massiliamazoniense* sp. nov., that contains strain ND2^T^ as type strain, in agreement with the results obtained by phenotypic, phylogenetic, and genomic characteristics. The strain was isolated from the fecal flora of a healthy 49-year-old Brazilian man.

## Taxonomic and Nomenclatural Proposals

### Description of *Clostridium massiliamazoniense* sp. nov.

*Clostridium massiliamazoniense* (ma.si.li.a.ma.zo.ni.e′n.se. N. L. gen. neutr. n. *massiliamazoniense*, a combination of Massilia, the Latin name of Marseille where strain ND2^T^ was first isolated and characterized, and Amazonia the origin of the patient from whom *C. massiliamazoniense* was first collected).

Colonies are dark gray with irregular edges and a diameter of 0.6 to 1 mm. Growth is observed at 37 and 45 °C in anaerobic atmosphere only, but is optimal at 37 °C. Cells are Gram-positive rods and have a length ranging from 1.90 to 3.0 µm and a width of 0.8 to 1.0 µm. They are not motile and form subterminal spores.

Catalase and oxidase negative. Indole is produced and nitrate is not reduced. Positive reactions were obtained with esterase (C4), valine arylamidase, α-chymotrypsin, acid phosphatase, naphthol-AS-BI-phosphohydrolase, β-galactosidase, alkaline phosphatase, d-glucose, and d-maltose. The type strain ND2^T^ (= CSUR P1360 = DSM 27309) was isolated from the fecal flora of a healthy 49-year-old Brazilian male living in the Amazonian part of Brazil. The major fatty acids were octadecanoic acid (14.4%) and hexadecanoic acid (12.9%). The genome is 3,732,600 bp long, with a G+C content of 27.6%. The 16S rRNA and genome sequences were deposited in GenBank under accession numbers HG315672 and CYSO00000000, respectively.
